# Comparative genomics provides new insights into the remarkable adaptations of the African wild dog (*Lycaon pictus*)

**DOI:** 10.1038/s41598-019-44772-5

**Published:** 2019-06-06

**Authors:** Daniel E. Chavez, Ilan Gronau, Taylor Hains, Sergei Kliver, Klaus-Peter Koepfli, Robert K. Wayne

**Affiliations:** 1 Department of Ecology and Evolutionary Biology, University of California, Los Angeles, California 90095 USA; 2 Efi Arazi School of Computer Science, Herzliya Interdisciplinary Center (IDC), Herzliya, 46150 Israel; 3 Environmental Science and Policy, Johns Hopkins University, Washington, D.C. 20036 USA; 4 Institute of Molecular and Cellular Biology, Novosibirsk, 630090 Russian Federation; 5 Smithsonian Conservation Biology Institute, National Zoological Park, Washington, D.C. 20008 USA; 6 Theodosius Dobzhansky Center for Genome Bioinformatics, Saint Petersburg State University, Saint Petersburg, 199034 Russian Federation

**Keywords:** Molecular evolution, Comparative genomics, Bioinformatics

## Abstract

Within the Canidae, the African wild dog (*Lycaon pictus*) is the most specialized with regards to cursorial adaptations (specialized for running), having only four digits on their forefeet. In addition, this species is one of the few canids considered to be an obligate meat-eater, possessing a robust dentition for taking down large prey, and displays one of the most variable coat colorations amongst mammals. Here, we used comparative genomic analysis to investigate the evolutionary history and genetic basis for adaptations associated with cursoriality, hypercanivory, and coat color variation in African wild dogs. Genome-wide scans revealed unique amino acid deletions that suggest a mode of evolutionary digit loss through expanded apoptosis in the developing first digit. African wild dog-specific signals of positive selection also uncovered a putative mechanism of molar cusp modification through changes in genes associated with the sonic hedgehog (SHH) signaling pathway, required for spatial patterning of teeth, and three genes associated with pigmentation. Divergence time analyses suggest the suite of genomic changes we identified evolved ~1.7 Mya, coinciding with the diversification of large-bodied ungulates. Our results show that comparative genomics is a powerful tool for identifying the genetic basis of evolutionary changes in Canidae.

## Introduction

**Figure 1:**
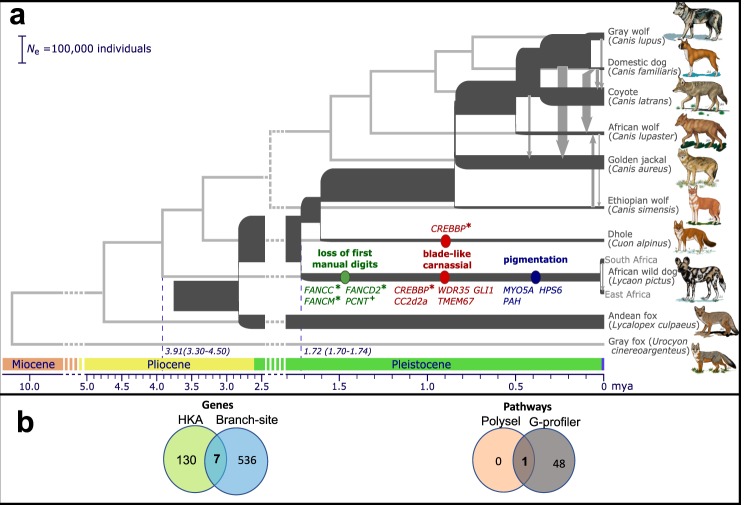
Evolutionary history and adaptation in the African wild dog (AWD) and nine other species of canids. (**a**) A species tree was inferred by applying ASTRAL-III to 8,117 25 kb-windows (light gray, in background), with internal nodes placed according to average genomic divergence estimated via MCMCTree and calibrated using two fossil priors (see Methods and Fig. S1 for details). A demographic model was inferred for the same species (excluding gray fox) by applying G-PhoCS to 11,112 putative neutral 1 kb windows (dark gray, in foreground). The same phylogenetic tree topology was assumed, augmented with 44 directed migration bands (see Methods and Table S2 for details). Block arrows depict the eight migration bands inferred with total rates higher than 0.05, with arrow widths scaled proportionally to the estimated total rate. The widths of branches in the demographic model are scaled proportionally to inferred effective population sizes (see scale bar at top-left), and their lengths are scaled proportionally to inferred species divergence times. Both scales assume an average per-generation mutation rate of μ = 4.0 × 10^−9^ and an average generation time of three years^88^. Species divergence times are much smaller than the average genomic divergence times. Divergence times associated with AWD are indicated at the bottom with 95% Bayesian credible intervals (note the change in time scale between 2.5–5 Mya). Genes with signals of positive selection are specified on the branches leading to the AWD and the dhole. Different phenotypic categories are indicated by color; genes marked with an asterisk had in-frame deletions and genes marked with a cross were pseudogenized. Note that *CREBBP* has undergone parallel adaptation in both lineages. (**b**) Venn diagrams showing shared positively-selected genes (left) and pathways (right) obtained from different analytical approaches. Among the seven genes that resulted in significant scores from both the HKA-like and branch-site tests, only *HPS6* was associated with AWD adaptations. Primary cilium was the only pathway that was identified by both G-profiler and polysel.

Among the living species of Canidae, the African wild dog (hereafter, AWD) is considered to be the most specialized with regard to adaptations for cursoriality, diet, and coat coloration^1^. Along with a gracile appendicular skeleton, the most notable characteristic of AWDs is the loss of the first digit on the forefeet. This trait increases their stride length and speed allowing them to pursue prey for long distances in open plain habitats and is unique among living canids^2^. The dentition of the AWD is also exceptional, as the teeth are generally sectorial in shape and the premolars are the largest relative to body size of any living carnivoran except spotted hyenas^3^. AWDs also show a transformation of the talonid on the lower first molar (carnassial) from a basin-like crushing depression into a trenchant heel or cutting blade for slicing flesh, which also occurs independently in two other hypercarnivorous canids, the bush dog (*Speothos venaticus*) and the dhole (*Cuon alpinus*). This feature is accompanied by the reduction or loss of post-carnassial molars, also a characteristic of hypercarnivorous canids^4^. Fossil evidence suggests that the reduction of the first digit and transformation of the carnassial in AWDs evolved gradually during the Plio-Pleistocene^5^. Finally, AWDs exhibit one of the most variegated coats among mammals, with individuals uniquely differing in pigmentation pattern and color^6^ (Fig. 1), which suggests the expression of a diversity of genes. The function of this highly individualistic coat pattern is uncertain but may represent an adaptation for concealment, communication or thermoregulation^7–10^.

We investigated the genetic origins of adaptations associated with the evolution of cursoriality, hypercarnivory, and coat color variation in the AWD in the context of their evolutionary and demographic history. To accomplish these goals, we sequenced one high coverage AWD genome and utilized three previously reported AWD genomes^11,12^. Additionally, we used three *de novo* AWD reference genomes^13^. Coverage depths are provided in the Supplementary Table S1. These genomes were compared with existing genomes from *Canis* (wolves, coyote and golden jackal) and *Cuon alpinus* (dhole)^11,12,14–18^. The genome of the bush dog was not included in this study because it is part of an ongoing research investigation on comparative genomics of South American canids^13^. We hypothesized that genes showing signals of positive selection and other molecular changes in AWDs are associated with digit reduction, tooth morphology, and pigmentation. Furthermore, we aimed to investigate the possibility of convergent evolution at the genetic level, exploring shared signals of selection among the wolf-like canids that have a trenchant heel (AWDs and dholes).

## Results and Discussion

### Evolutionary history

To provide an accurate evolutionary framework for the comparative genomic analyses of AWDs relative to other wolf-like canids, we first reconstructed the phylogenetic relationships among species of *Canis*, *Cuon*, and *Lycaon*. Species tree analysis using ASTRAL-III^19^ produced 102 distinct gene trees from 8,117 25 kb alignments sampled from 38 autosomes (~203 Mb), with the final species tree being the consensus topology of the 100 replicates from the analysis (Fig. 1). The resulting topology shows the Ethiopian wolf basal relative to the rest of *Canis*, with dhole and AWDs as successive sister lineages having diverged earlier (Fig. 1). Within *Canis*, the golden jackal and African wolf are independent lineages sister to the clade comprised of the coyote, gray wolf and domestic dog. These patterns of relationship are consistent with previous analyses based on nuclear DNA sequences^12,15,20^.

We next estimated the age of divergence of the AWD lineage using the inferred tree and two methods. First, we computed average genomic divergence times using MCMCTree^21^, in which two fossil priors were used to calibrate nodes of the phylogeny (see Methods for details). Our estimates suggest an average genomic divergence of 3.91 mya (95% HPD = 3.30–4.50 mya) between *Lycaon* and the clade containing *Cuon* and *Canis*, approximately two million years earlier than the earliest fossil evidence recorded for the lineage^1^. This more ancient divergence is feasible because average genomic divergence captures not only the time since species divergence, but also the time for lineages to coalesce in ancestral populations, which are known to be very large in canids^18^. To better address this discrepancy, we thus jointly inferred a complete demographic model using G-PhoCS^22^, taking into consideration gene flow between 44 possible pairs of branches in the phylogeny (see Methods for details). Indeed, the species divergence time for *Lycaon* is then estimated at 1.72 mya (95% HPD = 1.70–1.74 mya; Table S2 and Fig. 1), which is much closer to estimates from both the fossil record and recent analyses of whole-genome data^1,12,17^. Importantly, while our inferred model suggests prevalent gene flow between divergent canid species, *Lycaon* is inferred to be largely isolated from genetic exchange with other canid lineages. This isolation provided more time for unique genomic adaptations to evolve.

### African wild dogs are uniquely enriched in positively-selected genes related to primary cilia

To identify positive selection events that occurred on protein-coding genes during the evolution of the AWD lineage, the sequencing reads for four AWDs and eight other canid species were mapped to the domestic dog reference assembly (CanFam3.1) to take advantage of the high-quality annotation of the dog reference genome (Table S1). The mapping process was based on the GATK Best Practices pipeline (Methods). For almost all canids, we found that more than 97% of reads successfully mapped to the dog genome. The only exception was a low coverage (12.1x) AWD that had ~93% of the reads mapped to the dog. To avoid potential reference bias from aligning reads to a different species, we further confirmed our results on three recently published *de novo* AWD reference genomes^13^.

After calling genotypes with SAMtools and filtering with GATK 3.7^23^ as well custom python scripts, we identified ~19,000 orthologous protein-coding genes. Among these genes, 18,327 passed our quality filters (no internal stop codon, permissible length, and longest transcript) and were used to identify genes under positive selection using the branch-site model^21^. This test was conducted on each multi-species gene alignment generated with PRANK v.150803^24^ and using the topology in Fig. 1 as the guide tree. AWD, dhole, and gray wolf were specified as different foreground branches. A gene was considered positively selected if the value obtained from the likelihood-ratio test comparing a model where the ratio of nonsynonymous substitutions (d*N*) to synonymous substitutions (d*S*) was greater than 1 (d*N*/d*S* > 1) against a null model where d*N*/d*S* = 1. Significant differences were determinated with a chi-square distribution with 1 degree of freedom^25^.

One issue with the branch-site model is that it is highly sensitive to alignment errors. Therefore, we conducted an extensive filtering process on our data, first using SWAMP^26^ and then visually inspecting the alignments of genes with *p* < 0.05. After masking regions with unusual enrichment of amino acid changes, we conducted three independent runs for each foreground branch and gene family, and retained the one with the best likelihood-ratio score of each run^27^. This guaranteed that large log-likelihood ratios depicted from the branch-site model were not the result of convergence problems of the test^27^. Another concern in the exploration for genes under positive selection is the role of multiple nucleotide changes^28^. Although these changes may occur simultaneously, the branch-site model assumes that they occur in a successive manner. The result will be unrealistically high likelihood-ratio scores at a codon were nucleotide changes occurred at the same time^28^. Among our 12 candidate genes (Fig. 1), we identified three genes with multiple nucleotide changes (*CC2d2a*, *TMEM67*, *PAH*) and thus support for the positive selection on these genes should be interpreted with caution. Although it is challenging to elucidate the order of multiple nucleotide changes, our main conclusions are not affected even if we take a conservative approach and do not include such genes in the analysis.

We found 43 genes (Table S3) that were significant at a false discovery rate (FDR) of 20%, after conducting multiple hypothesis testing of 18,327 genes along the three foreground branches (AWD, dhole, and gray wolf). Since only a few genes passed the genome-wide significance threshold, we used all genes with a p-value ≤ 0.01 to test for enrichment of gene functions with G-profiler^29^. Ensembl identification of genes with a p-value ≤ 0.01 were input as query lists and the 18,327 total gene set was used as the background list. We allowed a minimum of two genes to overlap between query genes and genes belonging to a gene ontology (GO) term. This resulted in genes with specific signals of selection in AWDs overrepresented in terms related to primary cilia (Fig. 1), which are significantly involved in coordinating signaling pathways during mammalian development^30^.

The disadvantage of common tests for gene ontology enrichment like G-profiler^29^ is that an arbitrary significance cutoff must be specified, and data below that cutoff is expected to be lost. Therefore, instead of just focusing on some outlier genes with high likelihood ratios, we used polysel^31^ to conduct analyses of polygenic selection across the full set of tested genes. We looked for pathways that were overrepresented with genes having low or moderate likelihood ratios more than would be expected by chance. This model takes likelihood ratio test statistics estimated from the branch-site test and finds weak to moderate polygenic selection within biological pathways. Then, p-values are generated from an empirical null distribution obtained by randomly sampling gene sets in specified pathways. Using this approach, we also found that the “primary cilia” GO category was significantly enriched with a variety of levels of positive selection (Fig. 1b). To rule out the possibility that this GO category could be enriched just by chance due to the large number of genes, we tested for overrepresentation of significant genes in primary cilia in the gray wolf and dhole and found no evidence of enrichment. Finally, to account for possible errors generated from mapping short reads of the AWD to a different species (domestic dog), we verified every mutation reported in this study (e.g., nucleotide and amino acid deletions and substitutions) with a consensus sequence of the three recently published *de novo* AWD reference genomes (NCBI Bioproject PRJNA488046; Table S1)^13^.

### Digit reduction through apoptosis

Two developmental mechanisms of digit reduction from the ancestral five-digit morphology have been characterized in mammals. One is related to a complete absence of a digit during development through regulation of the transduction of sonic hedgehog (SHH) signaling and the other involves apoptosis of digits during early development^32^. The loss of the first digit, as found in AWDs, has been shown to be independent of SHH signaling^33^. Therefore, we focused our analyses on genes associated with apoptosis pathways, particularly those related to digit development.

We used the Variant Effect Predictor annotation tool^34^ to identify amino acid-changing substitutions unique to the AWD that could have a significant impact on the associated proteins but will be ignored by the branch-site model test. We identified 403 genes with both high and moderate impact. High impact indicates a disruptive substitution that could cause truncation, loss of function, or nonsense-mediated decay of a protein whereas moderate impact indicating a non-disruptive substitution that might change protein functional efficiency. The substitutions we identified were categorized as in-frame indels, frameshift variations, and stop codon gains (see Methods for details).

Strikingly, we found 596 genes with in-frame-deletions, with moderate impact, unique to AWDs. These amino acid deletions were tested for enrichment of GO categories using G-profiler^29^. We found that this type of mutation was overrepresented in digit-loss categories with a false discovery rate of 5%. Specifically, the term, “abnormality of the thumb,” was over-represented by the genes *FANCC*, *FANCD2*, and *FANCM*, which are associated with the Fanconi anemia (FA) pathway^35^. We also observed an overrepresentation of 33 genes with frameshift variation mutations in the olfactory receptor (OR) GO category. Twenty eight of these genes were also enriched in the olfactory transduction KEGG category, in accordance with the dynamic evolution of OR gene families^36^.

**Figure 2:**
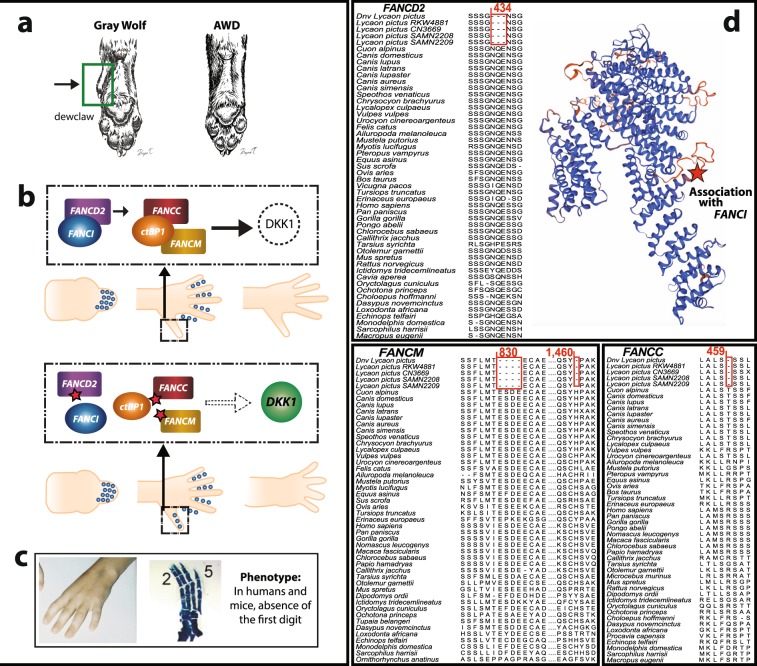
Apoptosis of the first digit in the African wild dog. **(a)** Primitive condition of five digits in the Canidae; note the small first digit in gray wolf called the “dewclaw”. The absence of the first digit is shown in the African wild dog (AWD). **(b)** Schematic representation of digit reduction and separation of digits. In the normal five-digit pattern scenario shown at the top, apoptosis (blue circles) is restricted to interdigital regions. The first digit, enclosed by a rectangle, is protected from apoptosis by FANCC and FANCM that form a complex with CtBP1 and repress DKK1^39^. The stability of this complex is regulated by the FANCD2-FANCI association^100^. In the scenario shown below, amino acid deletions (red stars) observed in FA genes may reduce both the affinity of FA genes to CtBP1 and the stability of the protein complex. Consequently, the FANCC-FNACM-CtBP1 complex is not formed and DKK1 is not suppressed (indicated by empty arrow). Deficiency of the FA complex activity increases DKK1 expression. As a result, apoptosis expands to the first digit; note blue circles (apoptosis) on the region of the thumb**. (c)** Effect of the mutations in the FA and *DKK1* genes in humans (Reprinted from ref.^35^© 2009 with permission from Elsevier) and mice (Reprinted from ref.^41^ © 2004 with permission from Elsevier). **(d)** Multiple sequence alignments of mammalian *FANCD2*, *FANCM*, and *FANCC* amino acid sequences showing deletions specific to AWDs. Five AWDs are shown; “RWK481” and “SAMN04312208” are individuals from Kruger National Park, South Africa; “CN3669” and “SAMN04312209” are individuals from Kenya and “*Dnv Lycaon pictus*” is the consensus sequence of three *de novo* reference AWD genomes^13^. The top panel also shows a 3D protein-model of FANCD2 with the location of the observed amino acid deletion, which is important for the association with FANCI.

Our results implicate amino acid deletions in genes associated with the FA pathway in the loss of the first digit in AWDs through an apoptosis pathway that typically directs interdigital cell death (Fig. 2). Specifically, in the development of the ancestral five-digit foot, the primary function of apoptosis is to eliminate excessive cells on the interdigital webs which trims the dimension of the digit^32,37^. When this digit individualization occurs during development, apoptosis has only a small effect on digit dimension^38^. Studies have shown that FA proteins form a complex with the CtBP1 protein that results in the repression of the *DKK1* gene. As expression of this gene is restricted to the interdigital area during the early development of digits, its repression prevents apoptosis from extending into the digits^37,39^. When the FA-CtBP1 complex fails to properly form, inhibition of *DKK1* expression is removed, thereby permitting apoptosis to extend to the digits. We suggest that the amino acid deletions in *FANCC*, *FANCD2*, and *FANCM* found exclusively in AWDs may reduce the binding affinity of the FA protein complex to CtBP1, thus allowing the loss of the first digit through apoptosis (Fig. 2). Our findings are strongly supported by the fact that the mutations we have identified in the FA genes in AWDs are responsible for a condition commonly associated with the absence of the first digit in humans^35,40^ and expression of *DKK1* is related with absence of the first digit in mice^41^. Moreover, apoptosis as a mode of evolutionary digit has also has been shown in horses, jerboas, and camels^32^.

### Hypercarnivory through sonic hedgehog (SHH) signaling

**Figure 3:**
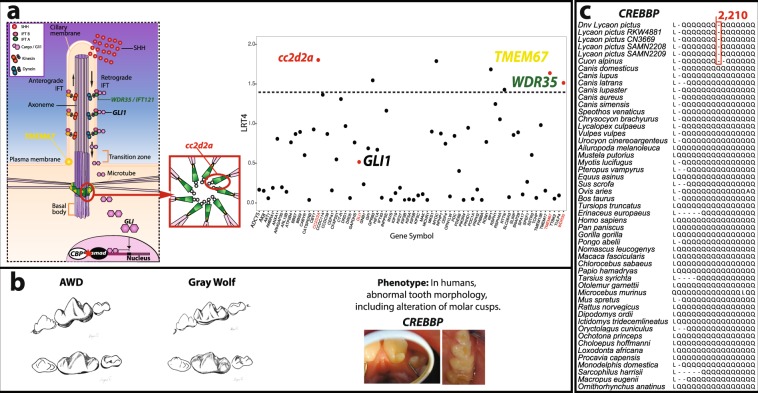
Possible mode of evolutionary molar cusp modification in the African wild dog through SHH signaling. **(a)** The figure at the top right shows genes found to be enriched in primary cilium with polysel; genes above dashed line are those found to be enriched with G-profiler as well (see Methods for details). Only gene names with known function in tooth development are shown. The figure at the top left shows a schematic of the primary cilium and the role of candidate genes in SHH transduction. SHH, represented by red circles, reaches the ciliary membrane. Then, transport of transcriptional factors GLIs such as GLI1, through the axoneme, is promoted by *WDR35/IFT121* as well as the efficient docking of the axoneme in the plasma membrane, which is conducted by *CC2d2a* and *TMEM67*. Together, these components of primary cilium cause rapid accumulation of GLI into the basal body. Ultimately, GLIs will enter the nucleus and promote the expression of SHH-dependent genes. In the case of *CREBBP*, the observed amino acid deletion (red star) may increase the affinity of this cofactor with smad genes and increase the expression of TGF-β and BMP dependent genes involved in molar cusp development. **(b)** Left-bottom figure showing the single cuspid talonid of the lower first molar (carnassial) in the AWD as opposed to a bi-cusped talonid carnassial in the gray wolf. Right-bottom figures show the effect that mutations in *CREBBP* have on humans^53^ (reprinted by permission from Wiley-Liss, Inc.; *American Journal of Medical Genetics*^53^ © 2007); talon cusp condition is observed; cusps that protrude from the anterior region of incisors on the left and extra cusps on molars on the right. **(c)** Amino acid alignment of the glutamine-rich region of *CREBBP* for 51 species of mammals showing the deletions specific to AWDs and the dhole. Five AWDs are shown; “RWK481” and “SAMN04312208” are individuals from South Africa; “CN3669” and “SAMN04312209” are individuals from East Africa; “*Dnv Lycaon pictus*” is the consensus sequence of three *de novo* reference AWD genomes^13^. The ancestral condition in canids is inferred as 15 glutamine residues.

The primary cilium is a hair-like structure that projects from the surface of cells and serves as a sensory organelle, transmitting signals from the extracellular space into the nucleus^42^ (Fig. 3a). This structure is located at the dental epithelium and mesenchyme during the formation of dental cusps^43,44^. As the primary cilium regulates numerous signaling pathways necessary for odontogenesis, ciliary defects can alter the process of cusp patterning^45^.

We found AWD-specific amino acid substitutions in components of the primary cilium, specifically the genes *WDR35*, *TMEM67*, *CC2d2a* and *GLI1*. These substitutions suggest a combined regulatory effect, through GLI transcription, on SHH-dependent genes (Fig. 3a). Specifically, the role of primary cilia depends on the retrograde (to the basal body region) and antegrade (to the tip of the ciliary membrane) intraflagellar transport (IFT) of the transcriptional factor GLI1^43,46^ (Fig. 3a). This transportation occurs through a microtubule structure called the axoneme. The efficacy of GLI mobilization through the axoneme is dependent upon protein-mediated transport by WDR35 (also known as IFT121) as well as the proper docking of the axoneme into the basal body region, which is mediated by TMEM67 and CC2d2a proteins^43,47,48^ (Fig. 3a). Mutations within genes encoding primary cilium components alter mobilization of GLI to the basal body, and hence result in gain or loss of GLI function in this region. An increase in GLI will result in the gain of SHH phenotypes such as growth of molar cusps. In contrast, a decrease in GLI will cause loss of SHH phenotypes^49^ such as inhibition of molar cusp development^44^. We suggest that the amino acid changes observed in AWDs in *WDR35*, *TMEM67*, *CC2d2a*, and *GLI1* may cause rapid transportation of GLI to the basal body, and consequently overexpression of SHH target genes^43,50^. Variants in the candidate genes reported in this study have been associated with abnormal tooth shape and may thus be related to the exaggerated hypercarnivorous dentition of AWDs, which includes the development of a lower trenchant carnassial^43,50,51^. Most of the amino acid sites from these genes were unique to AWD even when compared to other placental mammals and marsupials, including two South American canids, *Speothos venaticus* and *Chrysocyon brachyurus* (Chavez *et al*., unpublished data, Fig. S2). Also, specific sites in *CC2d2a* had a relatively high probability of being affected by positive selection as suggested by a Bayes empirical Bayes test (BEB > 0.90). Although nucleotide changes were not recorded for *GLI1* with BEB, mutations in this gene were located within significant windows as suggested by the $\frac{\theta }{D}$ estimate (p < 0.01; see Methods section) as estimated from the HKA-like test^52^, after verifying that the amount of information within windows of 25 kb in length was not driving higher differences between diversity and divergence (Fig. S3). Our results suggest that the transduction of SHH through primary cilium may have promoted the modification of a primitive molar with a posterior crushing basin into a trenchant sectorial single cusp in AWDs (Fig. 3b).

Another gene associated with spatial patterning of the tooth, *CREBBP*, was found to have an amino acid deletion in AWDs. Interestingly, we also observed a two amino acid deletion in the same region in the hypercarnivorous dhole, a canid that also possesses a lower trenchant carnassial heel (Fig. 3b)^4^. *CREBBP* is a strong candidate for the modified carnassial observed in these two hypercarnivorous canids. Notably, this gene is associated with abnormal numbers or features (talon cusps) of molar cusps in humans^53^ (Fig. 3b). Even though *CREBBP* is ubiquitously expressed, the shared amino acid deletions were in the glutamine-rich region of the protein (Fig. 3c). This region is highly conserved in eukaryotes and serves as the binding site for Smad proteins^54,55^. These proteins are transcriptional factors that regulate the expression of genes located at the dental lamina and mesenchyme, and play important roles in regulating differentiation and proliferation of cells during tooth development^56^. Specifically, Smad transcription factors enter the nucleus and bind to the coactivator *CREBBP* and regulate the expression of target genes (Fig. 3a). We suggest that the observed amino acid deletions observed in *CREBBP* in AWDs and dholes may alter the formation of the Smad-*CREBBP* complex. This ultimately will have a regulatory effect on the expression of TGF-β and BMP dependent genes^56^. In Smad knockout mice, dental cusp formation is affected^56^. Our results suggest that the formation of the trenchant heel of AWD, initially guided by primary cilium components, may be reinforced by a regulatory effect of Smad transcriptional factors on genes involved in tooth development^56^. Also, our findings suggest that this may be the regulatory pathway that also determines the blade-like cusps in the dhole.

The shared amino acid deletions in the *CREBBP* gene observed in AWDs and dholes (Fig. 3c) could have arisen through different routes. First, the changes could have evolved independently in each species. Alternatively, the changes could have resulted from past adaptive introgression between species, as has been found among species within the *Panthera* lineage^57^. To account for the latter possibility, we conducted tests of admixture in the context of the evolutionary and demographic history of the sampled canids^22^. Results from models with and without gene flow among different lineages suggested a history of extensive admixture among species of *Canis* (Supplementary Discussion; Table S2 and Fig. 1), consistent with recent findings^12^. However, the genomic data for AWDs and dholes suggest little or no gene flow between these lineages and those leading to *Canis* species. Although the lack of post-speciation gene flow between the dhole and AWD could suggest that the amino acid deleting changes in *CREBBP* evolved independently, we do not entirely rule out the possibility of shared post-divergence ancestry. Particularly, we found a low proportion of divergent sites between the AWD and dhole (only 9 divergent sites out of 3,000 flanking sites analyzed) around *CREBBP* that suggest a plausible shared ancestry. Regardless of the mode of evolution of *CREBBP*, the amino acid deletions shared between the AWD and the dhole may reflect similar selective forces favoring hypercarnivory^4^.

### Positively-selected genes associated with AWD pelage coloration

**Figure 4:**
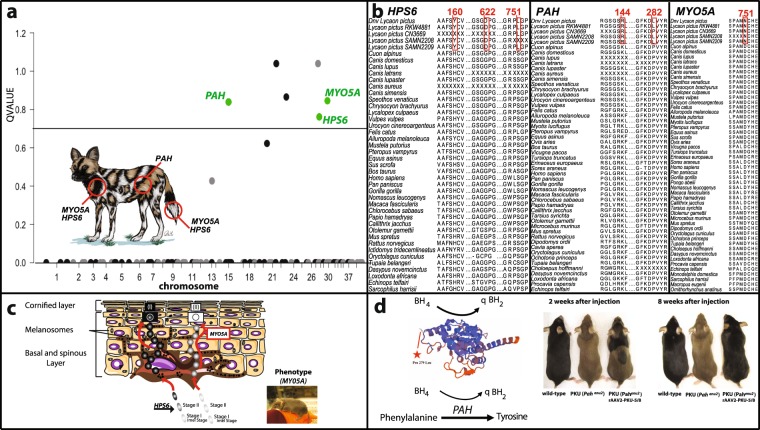
Candidate genes and possible mechanisms associated with coat pigmentation and patterning in African wild dogs. **(a)** Plot showing Q-values depicted from the branch-site test after a multiple hypothesis correction of 151 different coat color genes with the AWD as the foreground branch (see methods). Significant genes (Q-value < 0.20) are shown above the horizontal blue line; for illustration purposes, Q-values shown on the Y-axis were transformed to -log10. Only names of genes with relevant functions are shown. Illustration at the bottom showing coat color in the AWD; genes are shown in red circles and are placed on the type of color they regulate (e.g., *PAH* regulates brown and yellow colors). **(b)** Amino acid alignment of coat color candidate genes for an average of 49 species of mammals showing changes specific to AWDs. Five AWDs are shown: “RWK481” and “SAMN04312208” are individuals from Kruger National Park, South Africa; “CN3669” and “SAMN04312209” are individuals from Kenya and “*Dnv Lycaon pictus*” is the consensus sequence of three *de novo* reference AWD genomes^13^. **(c)** A schematic showing the role of candidate genes in color pattern. (I) shows that black coat color will need both proper cargo of melanin by HPS6 to melanosomes and its transport by MYO5A to the bulk of the hair. (II) In the case of white blotches or spots, they could be the result of either proper transport of melanosomes but containing no melanin or melanosomes containing melanin that fail to reach the bulk of the hair. The figure on the bottom-right shows the effect of *MYO5A* mutations on the pelage of a domestic mouse^69^. (**d)** 3D model of the PAH protein depicted with SWISS-model (see methods) that shows a mutation on the cofactor of the enzyme (B${{\rm{H}}}_{4}$). The scheme at the bottom illustrates the role of PAH and its cofactor. The right figure shows a gradual recovery of the black color of yellow mice (PKU) with deficiency of PAH^65^ (Reprinted by permission from Springer Nature: Springer Nature, *Gene Therapy* ref.^65^ © 2006).

Assuming that positive selection could have occurred in genes associated with the unique pelage coloration and patterning seen in AWDs, we tested a set of 151 genes that have been shown to be involved in mammalian pigmentation^58–62^ using the branch-site test^21^. We found six genes with AWD-specific signals of positive selection, three of which are known to have relevant function in coat coloration: *MYO5A*, *HPS6*, and *PAH* (Fig. 4a). The resulting amino acid changes were confirmed in a consensus sequence (“*Dnv Lycaon pictus*” in Fig. 4b) of three high-coverage *de novo* AWD genomes. Moreover, most amino acid changes in coat color were unique to AWDs when compared to other species of placental mammals, marsupials, and monotremes (Fig. 4b).

Considering that positively-selected substitutions related to the pigmentary system might fall outside protein-coding regions and could have also occurred during recent evolutionary history, we used the Hudson-Kreitman-Aguadé (HKA) test to examine regions with high divergence and low diversity for signals of selective sweeps. To conduct this test, we first called genotypes using Haplotype Caller in GATK 3.8^23^. We then calculated polymorphism within 100 kb windows among the four AWDs genomes that were mapped to the domestic dog reference genome. At the same time, per-site divergence was calculated between the AWD with the highest coverage (LPI_RKW 4881) and the domestic dog. Considering that demographic effects are expected to have an effect across the entire genome, we used empirical *p*-values to identify loci with extreme values of high divergence and low diversity as evidence for positive selection.

After verifying that the amount of information within 100 kb windows was not driving higher differences between diversity and divergence (Fig. S3), a total of 159 genes were located within windows with a magnitude of differentiation greater than expected by chance (empirical *p*-value < 0.01). Among the genes identified in the HKA test, seven were also observed in the branch-site test (Fig. 1). From this set of genes, we identified *HPS6* as a candidate gene that may have been recently selected. Among the set of genes with AWD-specific signals of positive selection, we did not observe four genes (*ASIP*, *MITF*, *MLPH*, *PMEL*) that were previously shown to have elevated ratios of non-synonymous/synonymous substitutions in two lower coverage AWD genomes^11^. For example, these authors reported a stop codon-gain in *PMEL*, but we found this was due to a misorientation in the codon translation frame (Fig. S4).

The positively-selected genes associated with pigmentation we detected have notable functions that strongly suggest regulation of the variegated pelage of AWDs (Fig. 4a). Although phenylalanine hydroxylase (*PAH*) has several pleiotropic effects, phenylalanine levels are closely related to melanin deposition^63–65^ (Fig. 4d). Levels of phenylalanine are determined by the *PAH* gene, whose protein catalyzes hydroxylation of phenylalanine to tyrosine^64,66^. Using available information to construct a 3D structure of the PAH protein, we located the AWD-specific mutation in the biopterin H domain (Fig. 4d), which contains a binding domain for the PAH cofactor^67^. This finding suggests that the mutation observed in the protein domain of *PAH* could be in part responsible for the AWD coat color pattern, as this gene and its cofactor are known to regulate the proportion of yellow and black fur^64^ through the conversion of phenylalanine to tyrosine^66^ (Fig. 4d). The observed amino acid change in *MYO5A* (myosin 5 A) is located at the myosin head region (Fig. S5), which is relevant to the motor function of the protein and the transportation of melanosomes^68^. Mutations in this gene result in patchy color patterns in mice^69^ (see Fig. 4c) and dilute pigment in dogs^61^. The AWD-specific mutations observed in *MYO5A* could be associated with white and black patches by regulating melanosome transport to the bulk of the hair^70,71^ (Fig. 4c). Similarly, sites under selection in *HPS6* may be responsible for the white and black patches by regulating melanin deposition in melanosomes^59,72^ (Fig. 4c). Mutations in this gene are associated with dilution of melanin in mice^72^ and Hermansky-Pudlak syndrome in humans (oculocutaneous albinism), a group of autosomal recessive disorders that cause abnormally light coloring of the hair and skin^73^. As all AWDs are born black^2^ and develop pigmentation patterns as puppies, longitudinal studies of gene expression will help corroborate the function of these genes in AWD pigmentation.

## Conclusions

A commonly observed trend among large mammalian predators that are cursorial is the elongation of limbs and the reduction or loss of digits which allows increased speed and improved pursuit and capture of increasingly faster prey^74^. Among canids, the AWD displays the most specialized morphological changes associated with cursoriality, including a unique reduction of the number of digits on the forepaws^75^. Similarly. AWDs have specialized carnassial molars that enhance the slicing of flesh. This decreases the consumption time of prey and therefore, the likelihood of encounters with competitors^4^. Also, while the function of the conspicuous and individual-specific coat coloration patterns of AWDs is uncertain, it may help confuse both prey and competing predators^76^, or it may not be the direct object of natural selection^77,78^.

Our comparative genomic analyses suggest that the evolution of cursoriality in AWDs has been driven by a known apoptotic pathway implicated in evolutionary digit loss in other mammals^32^ and involved a single major gene pathway. We also found evidence of substitutions and amino acid deletions in genes possibly associated with the hypercarnivorous dentition of AWDs and that changes in one of these genes (*CREBBP*) are also found in the Asiatic dhole. Consequently, our data support the idea that convergent phenotypic evolution can result from genetic changes in the same genes. Our study provides a unique example of genome-scale adaptive evolution analysis of one of the most successful pack-hunters, the African wild dog, and exemplifies molecular pathways which can iteratively adapt organisms to the challenges of prey capture and consumption.

The unique adaptations observed in African wild dogs were likely facilitated by their unique demographic history. Most large canid lineages have experienced gene flow from divergent species, whereas our inferred demographic model suggests that African wild dogs were genetically isolated from other species. Furthermore, divergence dating analyses provide a temporal framework for understanding the general rate of evolution of the molecular changes that underlie the morphological adaptations in AWDs. The earliest fossils of *Lycaon* (*L*. *sekowei* n. sp.) were described from sites in South Africa and dated to ca. 1.0–1.9 mya and suggest that the development of the hypercarnivorous dentition preceded the morphological changes associated with cursoriality in the modern AWD^1^. Divergence times estimated using a model that considers ancestral population size and post-divergence gene flow suggest that AWDs split from their common ancestor ~1.7 Mya, which is consistent with episodes of faunal turnover and the evolution of faster-moving ungulates during the Pleistocene that likely influence the adaptations of carnivores in sub-Saharan Africa^79,80^. Our study demonstrates that comparative analyses of genomes provide a powerful approach to investigate the genetic basis of unique adaptations in an evolutionary context.

## Methods

### DNA sample and sequencing

Genomic DNA from a female African wild dog (LPI_RKW 4881) was pair-end sequenced (100 bp) to ~27.9X coverage using an Illumina HiSeq2000 (Illumina, USA). The library preparation and genome sequencing was performed by the Vincent J. Coates Genomics Sequencing Laboratory at University of California, Berkeley. The individual that we sequenced originally belonged to the Skukuza pack in Kruger National Park, South Africa, and was originally identified as SF5^81^. This sample was selected based on sufficient quantities of high molecular weight DNA using a DNA fluorometer (Qubit 2.0), the NanoDrop spectrophotometer (ThermoFisher, USA) and gel electrophoresis. Genome sequences from 11 other canid species, including three African wild dogs, were obtained from previous studies^11,12,14–18^ and are detailed in Table S1.

### Alignment to the dog reference genome and annotation

An initial quality control of raw reads of LPI_RKW 4881, as well as those from 11 other canids obtained from the literature, was performed with FastQC (http://www.bioinformatics.babraham.ac.uk/projects/fastqc). Reads were then trimmed and filtered for adapters, short reads, and low-quality bases (Q < 20) with Trim Galore (http://www.bioinformatics.babraham.ac.uk/projects/trim_galore/)

before being aligned to the domestic dog genome reference assembly (canFam3.1) using Bowtie2^82^. The percentage of aligned reads to the domestic dog for most species was more than 97 and resulted in different coverage depths per species (Table S1). Variant calling was performed with HaplotypeCaller using the Genome Analysis Toolkit 3.7 (GATK)^23^ with a series of filtering steps to minimize the presence of false genotypes (Supplementary Methods).

### Species tree estimation

To reconstruct a phylogenetic tree of the the African wild dog and nine other species of canids, 8,177 sliding-window fragments of 25 kb were generated (Supplementary methods) and further aligned with PRANK v.150803^24^ using one iteration (-F once option) and the topology shown in Fig. 1a as the guide tree. Then, windows were trimmed using GBlocks^83^ with default parameters. Trimmed alignments were run with RAxML v8.2.9^84^ under the GTR model for each locus to infer individual maximum likelihood (ML) gene trees with 100 bootstrap replicates. For each locus, the best tree was selected from the RAxML output, while the 100 bootstrap trees were merged into a single file per locus. Additional alignment trimming and tree generation was done using a modified script from the SqCL pipeline^85^ (phylogeny_align_genetrees_prank.py). The best tree files were concatenated into one file with only 10% missing data tolerated, collapsing branch lengths shorter than 1e-05 substitution per site, and collapsing clades with support less than one using a script from the SqCL pipeline (phylogeny_prep_astrid_astral.py).

The species tree was estimated using ASTRAL-III v.5.5, which models the discordance between gene trees and species trees using a coalescent model^19^. We used both the concatenated best-tree and bootstrap tree files as inputs. The analysis was conducted with 100 bootstrap replicates and the best multi-locus tree was selected with ML support values. The best tree was then scored to obtain a posterior probability and quartet support values for each node/clade. The gray fox (*Urocyon cinereoargenteus*) was used as the outgroup to root the tree, based on results from previous molecular systematic investigations of canid relationships^20^.

### Estimation of divergence times

To estimate the ages of divergence among species of *Canis*, *Cuon*, and *Lycaon* (10 species, including the Andean fox and gray fox as outgroups) we first generated alignments for 1,183 single-copy coding orthologues. From the codon alignments, 166,182 four-fold degenerate sites (clock-like) were extracted and concatenated into a data matrix with 6,155 missing sites (3.7%) across the 10 species. Average genomic divergence times were estimated using MCMCTree from the software package PAML4^21^ with the HKY + G model of nucleotide substitution, the topology of the species tree obtained from the ASTRAL-III analyses as input, and 2,200,000 MCMC generations, of which the first 200,000 generations were discarded as burn-in. Other parameter settings used in the analysis are detailed in the Supplementary File. We applied two calibration priors with associated distributions and densities based on the fossil record of the Canidae to calibrate node ages, as previously described^14^. The first prior was set at the root with an age distribution of 9.0–11.9 million years ago (Mya), which provides an approximate age for the split between the tribes Canini and Vulpini, based on the first appearance of *Eucyon*, thought to be an early member of the Canini^86,87^. The second prior had an age range of 1.1–3.0 Mya ago, based on the earliest fossils of the modern gray wolf, *Canis lupus*, and the earliest known fossils of *Canis*, specifically, *Canis edwardsii*^86^.

Phylogenetic analyses suggest *Canis edwardsii* is sister to a clade that includes *Canis aureus* (golden jackal) and *Canis latrans* (coyote)^86^. However, since *Canis lupaster* (African wolf) and *Canis simensis* (Ethiopian wolf) are also contained in the genus *Canis*^12,15,20^, we assumed that the earliest known age of *Canis edwardsii* bracketed all extant species of *Canis*. The MCMC analysis was repeated twice, as recommended in MCMCTree manual, and no discordance was observed between runs.

### Demographic history and admixture

Twelve canid individuals were used in the demographic analysis, including the domestic dog reference and excluding the gray fox (see Table S1 for the remaining 11 genomes). Sequence alignments were obtained for these 12 genomes at 13,647 putatively neutral noncoding loci computed in previous studies to be short (1 kb long) interspersed (>30 kb apart) and distant from protein-coding genes (>10 kb)^18^. Of these alignments, 2,535 had more than 10% genotypes missing due to a sequencing depth below four reads or above the 95^th^ coverage percentile. These loci were removed and the remaining 11,112 loci were analyzed using the Generalized Phylogenetic Coalescent Sampler or G-PhoCS^22^. We assumed a population phylogeny consistent with the topology of the species tree inferred by ASTRAL-III. After labeling ancestral populations, we modeled gene flow by augmenting this phylogeny with 44 directional migration bands (Supplementary methods). An additional analysis was done assuming a species tree obtained by switching the position of the golden jackal and Ethiopian wolf in the species tree inferred by ASTRAL-III with the same 44 migration bands (see Supplementary Discussion).

We ran the multi-threaded version of G-PhoCS V1.3.2 (https://github.com/gphocs-dev/G-PhoCS) using five threads per run and a standard MCMC setup. Namely, we assumed an exponential distribution with mean of 0.0001 as the prior of all the mutation-scaled population sizes (θ) and divergence times (τ), and a Gamma (α = 0.002, β = 0.00001) distribution for the prior of migration rates (m). Because of the large number of migration bands, the Monte Carlo Markov chain was let to converge for 200,000 burn-in iterations, after which parameters were sampled every 50 iterations, for the next 400,000 iterations, resulting in a total of 8,000 samples from the approximate posterior distribution. For each parameter, we recorded the mean sampled value and the 95% Bayesian credible interval (CI). Population size estimates (Ne) were obtained from the mutation-scaled samples (θ) by assuming a mutation rate per-generation of μ = 4.0 × 10^−9^ ^88^, and divergence times (*T*) were calibrated by assuming the same rate and an average generation time of three years. Migration rates were scaled by the duration of time of the migration band, resulting in total rates, which approximate the probability that a lineage experienced migration. Parameter estimates are summarized in Table S2 and Fig. 1.

### Positive selection

Coordinates of ~19,000 orthologous genes were obtained using the domestic dog reference genome (canFam3.1) available in the Ensembl database^89^. To exclude paralogous genes, we filtered sites following previous recommendations^90^. Specifically, we filtered out sites according to the following criteria: (1) coverage higher than a 95^th^ percentile of distribution; (2) sites that occurred in more than one locus (with fix mate in GATK); and (3) duplicated sites likely generated from PCR libraries (with PCR duplicates in GATK). We also manually checked sequences for signals of duplication events and kept only sites that were bi-alellic.

To reduce the inclusion of false signals of positive selection caused by errors in the alignment process, short regions enriched with unreasonably high rates of nonsynonymous substitutions (dN) sites were masked with the Sliding Window Alignment Masker for PAML (SWAMP) tool^26^. Specifically, a two-step masking procedure was conducted. First, with dN ≥ 10 in a 15-codon window, followed by dN ≥ 3 in a 5-codon. This approach has been proven to effectively remove most of the problematic sequences associated with misalignment^26^.

Genes that passed our filters (no internal stop codon, permissible length and longest transcript) were then tested for signals of positive selection, based on the reconstructed species tree (Fig. 1a), using the branch-site model in PAML 4.8^21^ (Supplementary Methods). We run this model on the AWD and the other two pack-hunting species (dhole and gray wolf) were each used as foreground branches. Model A (allowing sites to be under positive selection; fix omega = 0) was compared to the null model A1 (sites may evolve neutrally or under purifying selection; fix omega = 1 and omega = 1). We included only genes with omega values > 1, since genes lower than this threshold could be driven by relaxed selection. Statistical significance (P < 0.05) was assessed using likelihood ratio tests (LRTs) and chi-square tests. Multiple hypothesis correction for 18,327 protein-coding genes and three foreground branches was conducted with a 20% false discovery rate (FDR) criterion using QVALUE in R^91^.

To detect recent signals of selection that include non-coding regions such as promoters and enhancers, we aimed to detect selective sweeps through an HKA-like approach^52,92,93^. To determine interspecific variation, genotypic variants were called with Haplotype Caller from the Genome Analysis Toolkit 3.8^23^. Independent gVCF files were created for the four AWD genomes (Table S1) and then joined with the option “CombineGVCFs” from GATK. On the multiple-samples gVCF, per-site polymorphism among the four AWDs was calculated across non-overlapping 100 kb windows with 10k steps between windows. At the same time, per-site divergence was calculated between the high coverage AWD (LPI_RKW 4881) and the domestic dog. These estimates were calculated with the following equation:

1 $$\frac{\theta }{D}=\frac{\frac{{{\rm{\Sigma }}}_{i=1}^{L}2{p}_{i}(1-p)}{L}\ast \frac{n}{n-1}}{\frac{{{\rm{\Sigma }}}_{i=1}^{L}{X}_{i}+0.5{Y}_{i}}{L}}$$

where *p* is the frequency of one allele in the four AWD genomes, *L* is the total number of callable sites with good quality in the window, *n* is number of sampled chromosomes (n = 8 for 4 diploid individuals), *X*_*i*_ is the number of derived alleles in the AWD (LPI_RKW 4881) with respect to the dog; and *Y*_*i*_ is the number of heterozygous sites.

Windows were required to have at least 10 kb of sequence and sites were filtered for low coverage (less than 3x and no more than a 95th percentile of distribution), low quality variant sites (QUAL < 50), missing genotype, non-bi-allelic sites, low quality genotype scores (Q < 20) and regions with high GC content. The windows with the lowest θ/D ratio were candidates for a selective sweep^92,94^. Empirical *p*-values were obtained for a total of 22,269 windows. To ensure that outliers were not driven by less sequencing data, we plotted the divergence and diversity ratio (θ/D) vs. the number of called bases per window (Fig. S3).

To further investigate candidate genes detected to be under positive selection, protein structure information from the relevant gene available in the literature was used. Nucleotide sequences of orthologous genes in FASTA format were translated into amino acids using “vespa.py translate” from the VEPSA tool^95^. The AWD protein sequences were then aligned to human annotated versions of the orthologous genes within Geneious v11.1.1^96^ to identify amino acid changes that occur within a given protein domain (see Fig. S4). When information was available, the effect of amino acid changes on protein structure and function was evaluated using three-dimensional models depicted from SWISS-MODEL^97^. Finally, to evaluate the degree of conservation (constraint) of the AWD-specific amino acid changes and deletions, AWD alignments were compared with available orthologous coding sequences from: (1) 12 other canid species, including corresponding sequences from the genomes *Speothos venaticus* (bush dog) and *Chrysocyon brachyurus* (maned wolf) (unpublished data); and (2) 29 to 41 other species of placental, marsupial and/or monotreme mammals, using the tool OrthoMaM v9^98^. Some amino acid changes in the *PAH* as well as the *WDR35* genes described in the result section were not observed in all samples of AWDs. However, they were confirmed on a consensus sequences of three high-coverage *de novo* ADW reference genomes (*“Dnv Lycaon pictus”* in Figs 4b and S2). This suggests that these mutations may be heterozygotes for *PAH* and *WDR35* genes, failing to be detected in some samples due to insufficient coverage.

### Enrichment test

Information about the functional impact of amino acid changes was obtained from the Ensembl Variant Effect Predictor tool^34^ with the domestic dog (Ensembl’s release89) and further used to identify indels (frameshift variation, in-frame insertions and in-frame deletions), loss-of-function mutations (stop codons) and regulatory variants (5^′^ UTR, 3^′^ UTR) that were unique to AWDs. Genes within these categories were then tested for enrichment of Gene Ontology (GO) categories using G-profiler version r1732_e89_eg36^29^, with a Benjamini-Hochberg (BH) false discovery rate (FDR) to correct for multiple testing.

To identify overrepresentation of genes with AWD specific-signals of positive selection depicted from the branch-site model, a GO analysis using G-profiler version r1732_e89_eg36^29^ was performed. Ensembl identification numbers of genes that resulted from the branch-site model (p < 0.05) were input as query lists, and all the genes tested (18,327 filtered genes) were employed as the background gene list. Multiple hypothesis correction was conducted with the Benjamini-Hochberg FDR method^99^. Likewise, Ensembl identification numbers of genes within windows that had empirical *p*-values < 0.01 from the HKA-like test were input as query lists, and all the genes tested were used as the background gene list.

### Polygenic selection

To detect biological pathways overrepresented by weak to moderate signals of selection on the AWD, dhole, and gray wolf, the program polysel (Detection of polygenic selection in gene sets) was employed^31^. Polysel uses information from all genes depicted by the branch-site model test to find low-level polygenic selection across many genes within a pathway. Significant pathways were identified with an FDR < 0.20, after removal of the overlap between pathways with the pruning procedure implemented in polysel (see Supplementary methods for details).

### Accession codes

A description of all custom scripts used for the analyses in this paper can be found at https://github.com/dechavezv/African_wild_dog_Project.git.

## Supplementary information

Supplementary information

## Data Availability

Numbers for the sequencing reads of canids genomes are listed in Table S1. The sequences of the red fox were obtained from BioMart - Ensembl (Ensembl Genes 95 release). *Speothos venaticus* (bush dog) and *Chrysocyon brachyurus* (maned wolf) sequences are available upon request from the authors. Gene sequences of other species of placental, marsupial and/or monotreme mammals, were obtained from OrthoMaM v9^98^.
