# Supplementary material for: Comparative genomics provides new insights into the remarkable adaptations of the African wild dog (Lycaon pictus)
Source: Sci Rep. 2019 Jun 6;9:8329. doi: 10.1038/s41598-019-44772-5 (PMC6554312; doi:10.1038/s41598-019-44772-5)
Supplement: Supplementary file 1 — Supplementary information [file 41598_2019_44772_MOESM1_ESM.pdf]

Supplementary Information

Comparative genomics provides new insights into the remarkable adaptations of the African wild dog (*Lycaon pictus*)

Daniel E. Chavez*[1], Ilan Gronau[2], Taylor Hains[3], Sergei Kliver[4], Klaus-Peter Koepfli[5,6], and Robert K. Wayne[1]

[1]Department of Ecology and Evolutionary Biology, University of California, Los Angeles, California, 90095 USA

[2]Efi Arazi School of Computer Science, Herzliya Interdisciplinary Center (IDC), Herzliya, 46150 Israel

[3]Environmental Science and Policy, Johns Hopkins University, Washington, D.C., 20036 USA

[4]Institute of Molecular and Cellular Biology, Novosibirsk, 630090 Russian Federation

[5]Smithsonian Conservation Biology Institute, National Zoological Park, Washington, D.C., 20008 USA

[6]Theodosius Dobzhansky Center for Genome Bioinformatics, Saint Petersburg State University, Saint Petersburg, 199034 Russian Federation

Supplementary Discussion

Demographic model with alternative topologies

According to our inferred demographic model, gene flow in AWDs is restricted to low rates between the two African populations (7.4%), with other analyzed species experiencing much higher rates of admixture. Most notable of these is the Eurasian golden jackal (*Canis aureus*), for which previous studies have also inferred high rates of ancestral gene flow [1,2]. Since the species tree inferred by ASTRAL-III does not account for gene flow, the location of the golden jackal branch could be falsely inferred as part of the clade containing all wolf species. To ensure that this uncertainty in species tree topology does not influence our estimates of demographic parameters associated with AWDs or the dhole (*Cuon alpinus*), we inferred an alternative set of demographic parameters assuming a species tree in which the golden jackal is an outgroup to all wolf populations, including the Ethiopian wolf (obtained by switching the location of the golden jackal and the Ethiopian wolf in the tree).

The estimates of demographic parameters obtained under this alternative model were highly concordant with our original estimates, with overlapping credible intervals for nearly all parameters (see Table S2). The main difference between the two inferred models related to the time period between divergence of the golden jackal and Ethiopian wolf from other populations (between T_{ANC5} and T_{ANC4}). Under the original model (based on the tree inferred by ASTRAL-III), the two species diverged from other lineages during a short period of time roughly 842,500 years ago, with a significant population size decrease during this brief period. However, under the alternative model, the golden jackal diverged 945,600 years ago and the Ethiopian wolf diverged 175,000 years later. This difference may indicate that the golden jackal diverged from other *Canis* lineages prior to the Ethiopian wolf, and genomic similarity between the golden

jackal and several wolf species is now partly explained by post-divergence gene flow. Importantly, this uncertainty in the topology of the species tree does not significantly influence any of the parameters associated with AWDs or the dhole.

Supplementary methods

Alignment to the dog reference genome and annotation

To conduct a comparison between canid genomes, reads were aligned to the domestic dog with Bowtie2[3]. Then, duplicates that may arise during library construction using PCR were removed with Picard tools 1.80 (https://broadinstitute.github.io/picard/). To correct mapping errors made by genome aligners and to ensure that reads were properly paired, local realignment around indels was conducted with the Genome Analysis Toolkit 3.7 (GATK) IndelRealigner tool[4]. To reduce potential errors introduced by sequencing, base quality scores were recalibrated with the GATK BaseRecalibrator tool. Bases at sites with expected variation were masked with "known variants", which were obtained by calling genotypes with three different tools: the GATK HaplotypeCaller, GATK UnifiedGenotyper and SAMtools mpileup[4,5]. Then, genotype calls that were found in two or more of these tools were extracted with BCFtools[5]. To ensure convergence between reported and empirical genotype quality scores, base score quality recalibration was run three times. Only reads that were calibrated, properly mapped and with high quality (Phred scores >= 20) were employed for further analysis.

To minimize the inclusion of erroneous genotypes, a series of filters were applied to called variants. Specifically, sites with a read depth less than 4 or greater than the 95th percentile of depth for each individual genome were filtered out. To reduce bias in downstream analysis

due to base composition heterogeneity, CpG islands were removed following previous studies[6]. To ensure the inclusion of variants that were both substitutions and bi-allelic, the following sites were removed from the original VCF files: indels, multi-nucleotide polymorphisms, and sites with more than one alternate allele. Finally, to conserve only high-quality reads, only variant sites with high quality scores (GQ≥30) were retained in the final VCF file.

Collection of multi-species sequence alignments

Most of the analyses conducted in this study require multi-species alignments in FASTA format. Therefore, whole-genomes in FASTA format were obtained from BAM files by first creating pileup files using SAMtools 1.2 (mpileup with -uv options)[5]. Pileup files were converted to VCF files with BCFtools 1.1-108-g1844401 (-c option), filtering out low-quality variants (Phred quality scores > 20, min depth coverage > 3x, and maximum depth coverage < 95th percentile of species total coverage). Filtered VCF files were converted to consensus FASTQ files using vcf2fq in vcfutils.pl from SAMtools, and further transformed into FASTA files with seqtk v.1.2 (https://github.com/lh3/seqtk/blob/master/seqtk.c). Once whole genomes in FASTA format were obtained, specific regions (e.g neutral regions, orthologs; see next sections) were extracted with BEDtools v2.26 (-getfasta option), (http://bedtools.readthedocs.io). Finally, the multi-species FASTA sequences were aligned with PRANK v.150803[7] using one iteration (-F once option) and the topology shown in Fig. 1a as the guide tree.

Species tree estimation

To generate 25kb genome fragments suitable for a phylogenetic reconstruction, we first called genotypic variants with Haplotype caller with GATK[4]. This generated independent gVCF files for 12 canid genomes (see Table S1) that were combined with "CombineGVCFs" tool from GATK. Then, in conjunction with SAMtools[5] and BEDtools (http://bedtools.readthedocs.io), a custom python script was written to generate non-overlapping sliding-window fragments of 25kb that mapped to the 38 canid autosomes. Window size was chosen based on previous studies of relationships among closely related species of carnivores[8]. Only windows with more than 700 informative sites were kept. A site was considered informative if at least nine genomes out of twelve had information about their genotype (i.e., either variant or invariant sites). In contrast, a site was considered not informative if more than 4 genomes had masking substitutes of low-quality base calls with 'N's (undetermined bases). This filtered out roughly 5% of the windows genome-wide. FASTA sequences were then obtained for the remaining overlapping windows.

Demographic History and Admixture

The demographic history model was conducted using the topology shown in Fig.1 along with ancestral populations that were label as follows:

- ANC1 – ancestral to domestic dog and gray wolf.
- ANC2 – ancestral to dog, gray wolf, and coyote.
- ANC3 – ancestral to dog, gray wolf, coyote, and African wolf.
- ANC4 – ancestral to dog, gray wolf, coyote, African wolf, and golden jackal.
- ANC5 – ancestral to the entire *Canis* clade (including Ethiopian wolf).

– ANC6 – ancestral to the *Canis* clade and dhole.
– AWD – ancestral to the two sampled AWD populations (from East and South Africa)
– ANC7 – ancestral to the *Canis* clade, dhole, and *AWD*.
– ROOT – ancestral to all sampled individuals, including the Andean fox.

Gene flow was modeled by augmenting the phylogeny of Fig.1 with the 44 directional migration bands:

– Thirteen migration bands between the golden jackal and wolf-like canids, as inferred in several previous studies[1,2]. Specifically, bands from dog to golden jackal and between the golden jackal and each of gray wolf, African wolf, dhole. Also, bands between the golden jackal and the three populations ancestral to both dog and gray wolf but not to Ethiopian wolf or golden jackal (ANC1, ANC2, and ANC3).
– Five migration bands between the coyote and other canids, as inferred by previous studies[9,10]: Bands from dog to coyote and between the coyote and either the gray wolf or ANC1 (the population directly ancestral to dog and gray wolf).
– Eighteen migration bands between AWD and other populations: Bands between the two sampled AWD populations. Also, bands between African wolf or dhole and each of the three AWD populations (including the one directly ancestral to the two sampled populations). Additionally, four bands from gray wolf or dog into each of the two AWD populations.

– Eight migration bands involving African wolf, Ethiopian wolf and dhole. Specifically, Bands from the dog and gray wolf to each of dhole, African wolf, and Ethiopian wolf. Also, bands between African wolf and Ethiopian wolf.

Positive Selection

To prepare files for the branch-site test of positive selection [11], a set of python scripts from the tool VESPA[12] were used. Only sequences that were confirmed as protein coding (length of the sequence divisible by 3), did not have internal stop codons, and were the longest transcript available, were kept for the positive selection analysis using "vespa.py clean". Sequences were further translated to amino acid alignments with "vespa.py translate" to ensure that no incomplete codons or internal stop codons were included. Amino acid sequences were aligned with PRANK v.150803[7] using one iteration (-F once option) and the topology shown in Fig. 1a as the guide tree. Then, a database with all amino acid sequences was created with "vespa.py create_database" to assist the next process of backward transformation from amino acid to the nucleotide sequence. The latter step was done with the command "vespa.py map_alignments". A directory structure for PAML analysis was created with a custom bash script. Each individual folder contained a multiple sequence alignment for a specific gene and a phylogenetic tree corresponding to Fig. 1a with the labeled foreground branch. Also, each folder included a control file with information about the null model (fixed Omega) and the model assuming positive selection ("free" Omega).

Polygenic selection

To conduct analyses of polygenic selection across the full set of tested genes in PAML, pathways were extracted from NCBI (https://www.ncbi.nlm.nih.gov/biosystems/), with the options "pathway"[BioSystemType] and "*Canis lupus familiaris*"[Organism]. Then, based on information available in the literature, a subset of pathways relevant to digit development, tooth formation, and pigmentation were chosen as input for polygenic signals associated with species adaptations[13-16]. Once the pathways were specified, likelihood ratio test statistics of all genes in each pathway were added in the form of 'SUMSTAT' scores. To estimate the 'SUMSTAT' scores, the ΔlnL4 score was calculated as the fourth root of the log-likelihood ratio previously obtained with the branch-site test for positive selection in PAML4[16]. Ensembl gene IDs were transformed into Entrez gene IDs using information within the gene2ensembl file from the NCBI ftp server (ftp://ftp.ncbi.nih.gov/gene/DATA/gene2ensembl.gz). Once a gene set and pathways were specified as input to polysel, a null distribution for each pathway was created by randomly sampling genes from the original gene set to generate pathways of a similar size as the ones provided in the input. Then, p-values were obtained by detecting how often a 'SUMSTAT' score exceeds those from the null distribution. Finally, a multiple testing correction was performed by first removing high-scoring genes from lower level pathways in a process called pruning in polysel. Then, p-values were re-calculated again from the pruned pathways. This process was conducted 300 times, creating an empirical FDR distribution. Significant pathways were identified with an FDR <0.20.

Supplementary Tables

Table S1. Basic sequencing statistics related to the genomes analyzed in this study. Statistics were calculated with Qualimap on BAM files after base quality recalibration.

Common name	Scientific name	Sample ID	Reference	Mean depth coverage (X)	Base Pairs (Gbp)	% of reference covered by at least 3 reads	Total number of reads aligned (x10^6)
**African wolf**	*Canis lupaster*	RKW1356	[17]	27.96	65	98.11	655
**Andean fox**	*Lycalopex culpaeus*	SRS523207+	[18]	10.98	25.5	97.71	264
**AWD East Africa**	*Lycaon pictus*	SAMN04312209*	[19]	12.1	28.1	93.94	146
**AWD East Africa**	*Lycaon pictus*	SAMN10180432* (CN3669)	[20]	17.28	40.2	98.3	521
**AWD South Africa**	*Lycaon pictus*	SAMN04312208*	[19]	10.67	24.8	96.66	126
**AWD South Africa**	*Lycaon pictus*	SAMN09924608*	This Study	27.93	65	98.71	674
**AWD East Africa**	*Lycaon pictus*	SAMN09917439[1]	[21]	69	NA	NA	NA
**AWD East Africa**	*Lycaon pictus*	SAMN09917479[1]	[21]	46	NA	NA	NA
**AWD - captive**	*Lycaon pictus*	SAMN09917480[1]	[21]	25	NA	NA	NA
**Coyote**	*Canis latrans*	RKW13455	[9]	25.67	59.7	98.72	604
**Dhole**	*Cuon alpinus*	SAMN10180424*	[20]	19.53	45.4	98.35	511
**Ethiopian wolf**	*Canis simensis*	SAMN10180425*	[20]	9.66	22.5	97.37	247
**Golden jackal**	*Canis aureus*	SAMN03366713* (RKW1332)	[1]	26.09	60.7	97.46	1102
**Gray fox**	*Urocyon cinereoargenteus*	SAMN04495241*	[22]	18.47	42.9	97.49	450
**Gray wolf**	*Canis lupus*	RKW1547	[2]	27.33	63.6	98.76	643

*BioSample IDs as shown in NCBI
+trace.ddb
[1]Genomes employed exclusively for validation of specific mutations.

Table S2. Demographic parameter estimates inferred by G-PhoCS. Analysis was done under a model with population phylogeny with the topology of the species tree inferred by ASTRAL-III and 44 migration bands (middle column) as well as a model in which the golden jackal and the Ethiopian wolf are switched in the population phylogeny (right column). Effective population sizes are calibrated by assuming an average per-site mutation rate of $\mu=4.0\times10^{-9}$ [23], and divergence times are calibrated assuming the same rate and an average generation time of three years. Total migration rates are obtained by multiplying the mutation-scaled rate with the duration of time of the migration band. For low rates, this approximates the probability that a lineage experienced migration. Total migration rates are shown for the 18 migration bands inferred by G-PhoCS to have the highest total migration rates. The total migration rates of the remaining 26 bands were estimated with mean value 0.0 and 95% Bayesian credible interval below 0.005 in the two runs. The six parameters for which the 95% Bayesian credible intervals from the two analyses do not overlap are shown in bold.

Parameter	Mean estimate (95% Bayesian CI)	
	Tree inferred by ASTRAL-III	Tree with Golden jackal and Ethiopian wolf switched
Population divergence times (years)		
T_{AWD}	26,000 (23,000-29,300)	25,700 (23,100-28,300)
T_{ANC1}	72,200 (66,700-77,800)	70,600 (63,800-81,700)
T_{ANC2}	362,500 (329,300-392,000)	349,800 (317,300-385,400)
T_{ANC3}	498,900 (478,100-520,400)	523,000 (492,500-565,000)
$\boldsymbol{T_{ANC4}}$	**842,500 (827,600-859,500)**	**770,700 (748,200-794,100)**
$\boldsymbol{T_{ANC5}}$	**842,700 (827,700-859,600)**	**945,600 (920,800-970,900)**
T_{ANC6}	1,610,200 (1,585,600-1,634,900)	1,597,200 (1,569,700-1,624,300)
T_{ANC7}	1,722,800 (1,703,900-1,741,600)	1,724,200 (1,705,300-1,742,700)
T_{ROOT}	2,823,400 (2,792,400-2,854,000)	2,823,200 (2,793,400-2,853,300)
Effective population sizes (number of individuals)		
Ne (AWD-SA)	16,400 (14,800-17,900)	15,700 (14,300-17,200)
Ne (AWD-EA)	19,300 (17,600-21,300)	20,100 (18,200-22,100)
Ne (AWD)	29,800 (29,000-30,600)	29,800 (29,000-30,600)
Ne (Gray wolf)	27,100 (25,100-29,300)	26,800 (24,200-30,100)
Ne (Coyote)	65,700 (61,700-69,800)	62,900 (58,600-67,200)
Ne (African wolf)	12,700 (11,700-13,700)	11,800 (10,500-13,000)
Ne (Golden jackal)	51,000 (49,000-53,200)	51,100 (49,000-53,200)
Ne (Ethiopian wolf)	9,100 (8,500-9,700)	9,600 (9,000-10,200)
Ne (Dhole)	11,200 (10,600-11,700)	11,200 (10,600-11,700)
Ne (Andean fox)	50,500 (49,100-51,900)	50,500 (49,100-52,000)
Ne (ANC1)	124,300 (115,500-132,700)	127,800 (117,400-136,600)
Ne (ANC2)	176,400 (140,200-210,300)	190,200 (162,100-220,800)
Ne (ANC3)	122,200 (114,400-130,800)	105,600 (88,300-118,700)
***Ne* (ANC4)**	**12,000 (1,600-35,600)**	**162,200 (138,100-186,100)**
Ne (ANC5)	119,700 (115,400-123,800)	111,400 (105,700-117,000)
Ne (ANC6)	92,200 (69,000-115,800)	105,600 (79,100-130,800)
Ne (ANC7)	166,600 (160,300-173,000)	166,200 (160,000-172,800)
Ne (ROOT)	108,700 (103,400-114,100)	108,700 (103,400-114,000)
Total migration rates		
m(ANC1→Golden jackal)	0.376 (0.306-0.474)	0.405 (0.333-0.469)
m(Dog→African wolf)	0.326 (0.299-0.354)	0.340 (0.307-0.378)

m(Dog→Coyote)	0.155 (0.116-0.192)	0.132 (0.083-0.177)
m(Ethiopian wolf→African wolf)	0.135 (0.124-0.148)	0.148 (0.134-0.163)
m(ANC2→Golden jackal)	0.111 (0.000-0.188)	0.242 (0.159-0.320)
m(Gray wolf→Coyote)	0.092 (0.064-0.129)	0.121 (0.091-0.158)
m(AWD-SA→AWD-EA)	0.074 (0.036-0.135)	0.028 (0.013-0.046)
***m*(African wolf→ Ethiopian wolf)**	**0.060 (0.045-0.073)**	**0.023 (0.011-0.036)**
m(Coyote→Gray wolf)	0.044 (0.028-0.057)	0.041 (0.025-0.058)
m(African wolf→Golden Jackal)	0.042 (0.033-0.052)	0.048 (0.036-0.058)
***m*(Coyote→ANC1)**	**0.041 (0.026-0.063)**	**0.002 (0.000-0.014)**
m(Golden jackal →Dhole)	0.030 (0.025-0.036)	0.033 (0.026-0.040)
m(Dog→ Golden jackal)	0.027 (0.006-0.045)	0.003 (0.000-0.017)
m(Dog→Ethiopian Wolf)	0.010 (0.006-0.014)	0.011 (0.008-0.014)
m(ANC1→Coyote)	0.003 (0.000-0.031)	0.027 (0.000-0.073)
m(Golden jackal→African Wolf)	0.002 (0.000-0.011)	0.001 (0.000-0.010)
m(ANC3→Golden jackal)	0.001 (0.000-0.012)	0.002 (0.000-0.014)
***m*(AWD-EA→WildDog-SA)**	**0.001 (0.000-0.007)**	**0.038 (0.020-0.062)**

Table S3. Genes detected to be under positive selection using the branch-site test in PAML4 and after multiple hypothesis-testing correction on three foreground branches (African wild dogs, dhole, and gray wolf) using 18,327 genes. Only genes significant for the branch of the African wild dog (AWD) and with qvalues < 0.20 are shown. Unavailable gene name is indicated by "N/A.

Ensembl ID	Gene symbol	P value	Q value
ENSCAFG00000014773	HSPG2	0	0
ENSCAFG00000015624	LEO1	8.33E-13	2.29E-08
ENSCAFG00000030852	N/A	8.33E-10	1.53E-05
ENSCAFG00000000070	PHLPP1	7.72E-09	0.000106117
ENSCAFG00000015593	N/A	9.24E-08	0.001016086
ENSCAFG00000004101	BICRA	1.53E-07	0.00136672
ENSCAFG00000006937	N/A	1.74E-07	0.00136672
ENSCAFG00000006714	NLRP14	2.39E-07	0.001642617
ENSCAFG00000005629	N/A	2.95E-07	0.001654988
ENSCAFG00000029964	N/A	3.01E-07	0.001654988
ENSCAFG00000003045	ZNF292	1.31E-06	0.005301932
ENSCAFG00000011482	PABPN1	1.35E-06	0.005301932
ENSCAFG00000016111	FOXK1	1.91E-06	0.007001169
ENSCAFG00000003025	RASSF6	2.13E-06	0.007277162
ENSCAFG00000007121	FEZF2	2.25E-06	0.007277162
ENSCAFG00000012783	NHS	2.50E-06	0.007636528
ENSCAFG00000012447	TRAF3IP1	3.70E-06	0.010707216
ENSCAFG00000024733	N/A	4.52E-06	0.012426158
ENSCAFG00000009552	IMPG2	4.92E-06	0.012881731
ENSCAFG00000023991	N/A	6.50E-06	0.016244977
ENSCAFG00000017716	EEF2K	8.38E-06	0.020032937
ENSCAFG00000007213	CHPT1	1.04E-05	0.023825967
ENSCAFG00000001545	TTC39B	1.09E-05	0.023972588
ENSCAFG00000031056	MANEA	1.25E-05	0.025455093
ENSCAFG00000029006	N/A	1.25E-05	0.025455093
ENSCAFG00000005441	DLL3	1.84E-05	0.036131686
ENSCAFG00000001554	BOP1	3.10E-05	0.058774931
ENSCAFG00000011856	N/A	3.27E-05	0.05993147
ENSCAFG00000003816	SMPD2	5.06E-05	0.089746445
ENSCAFG00000015595	IGSF21	5.71E-05	0.096381965
ENSCAFG00000000938	MCM9	5.94E-05	0.096381965
ENSCAFG00000005330	SH2D4A	5.96E-05	0.096381965
ENSCAFG00000016793	ZNF646	6.47E-05	0.101640003
ENSCAFG00000001178	COL22A1	7.16E-05	0.109355078
ENSCAFG00000004611	GRIN2C	9.37E-05	0.139240732
ENSCAFG00000011155	STARD9	0.000105578	0.151227217
ENSCAFG00000031292	NKX1-2	0.000107267	0.151227217
ENSCAFG00000029563	DIO3	0.000121385	0.166852786
ENSCAFG00000032438	N/A	0.000139159	0.186619007
ENSCAFG00000031975	N/A	0.000144209	0.188786749
ENSCAFG00000025358	KRT72	0.000156208	0.198060984
ENSCAFG00000011046	BCL9	0.000160622	0.198060984
ENSCAFG00000024785	ANKRD40	0.0001621	0.198060984

Supplementary Figures

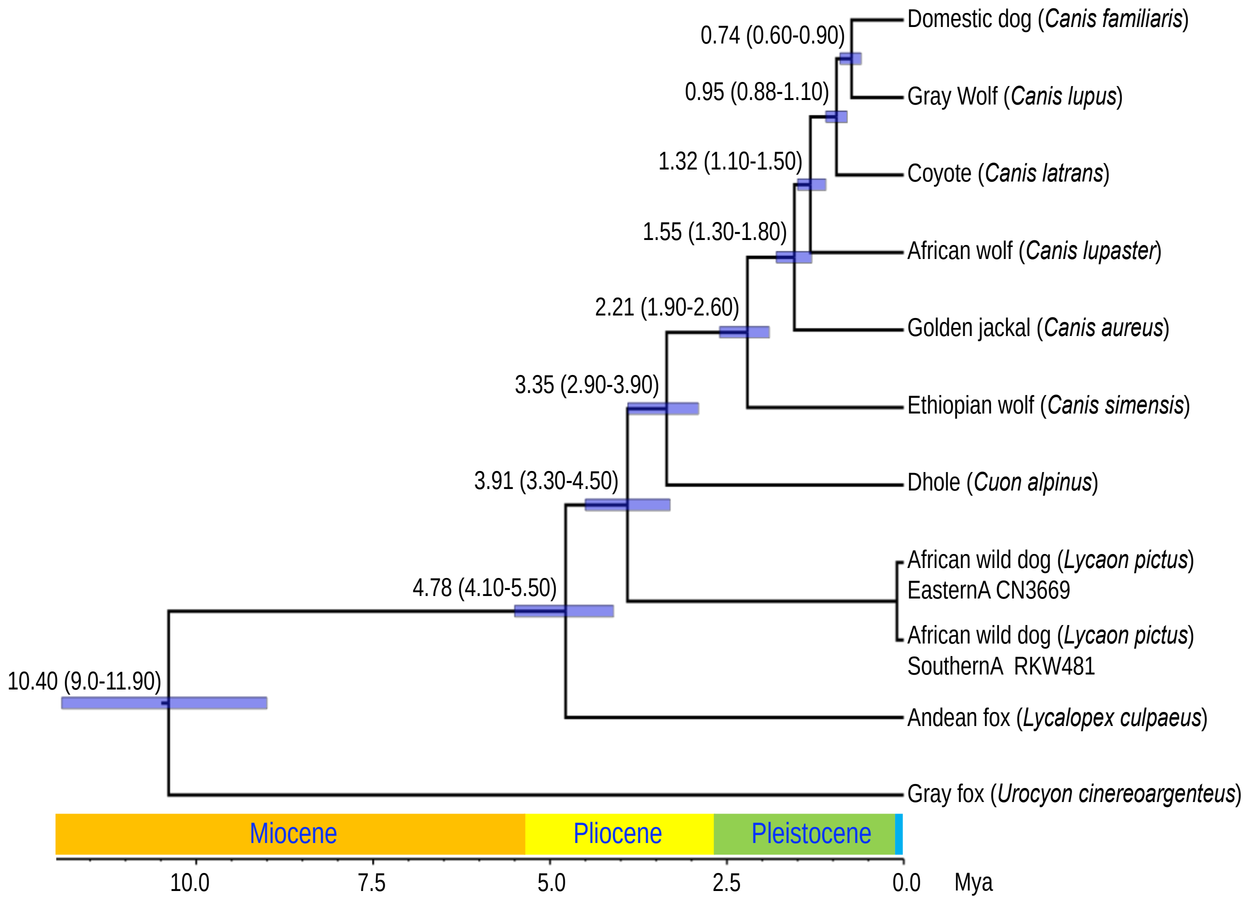

Figure S1. Coalescent species tree inferred by applying ASTRAL-III to 8,117 25kb-windows with internal nodes placed according to average genomic divergence estimated via MCMCTree and calibrated using two fossil priors (see Methods for details).

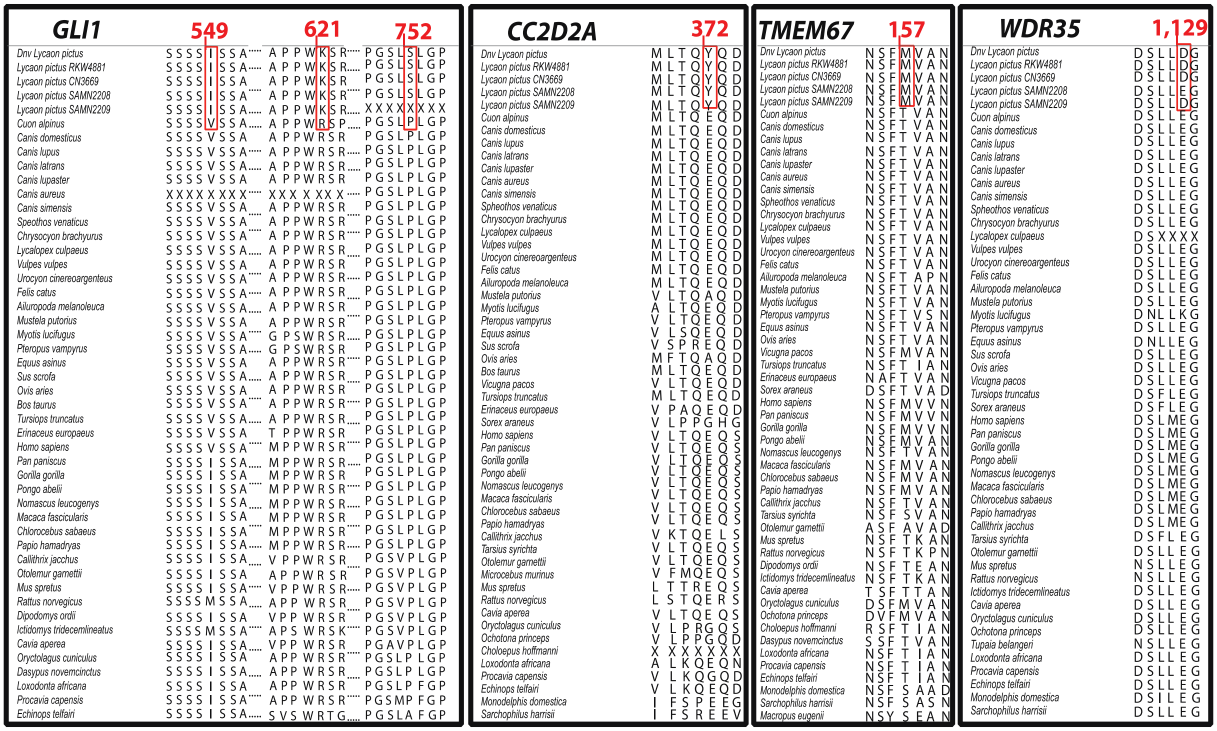

Figure S2. Multiple sequence alignment of mammalian genes that are candidates for blade-like molar cusps in AWDs. Amino acid changes of AWD (*Lycaon pictus*) are enclosed in red rectangles. Five sequences of AWD are shown; "RWK481" and "SAMN04312208" are individuals from Kruger National Park, South Africa; "CN3669" and "SAMN04312209" are individuals from Kenya and "Dnv_*Lycaon pictus*" is the consensus of the tree *de novo* reference AWD genome

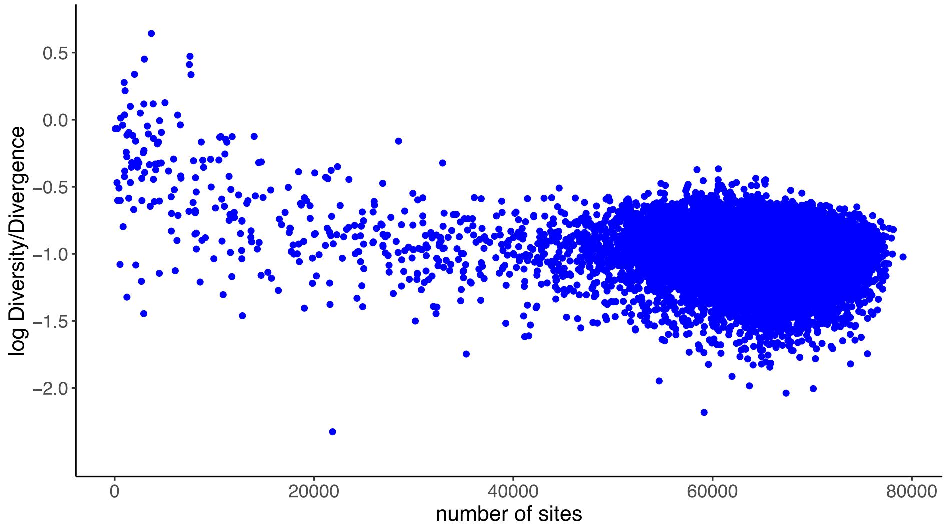

Figure S3. The relationship between the ratio of diversity/divergence and amount of genotype calls (after filtering) within 100-kb windows. Each point represent a window; diversity was calculated among four African wild dog samples, and divergence was calculated by comparison of the AWD (LPI_RKW 4881) with the domestic dog (see Methods).

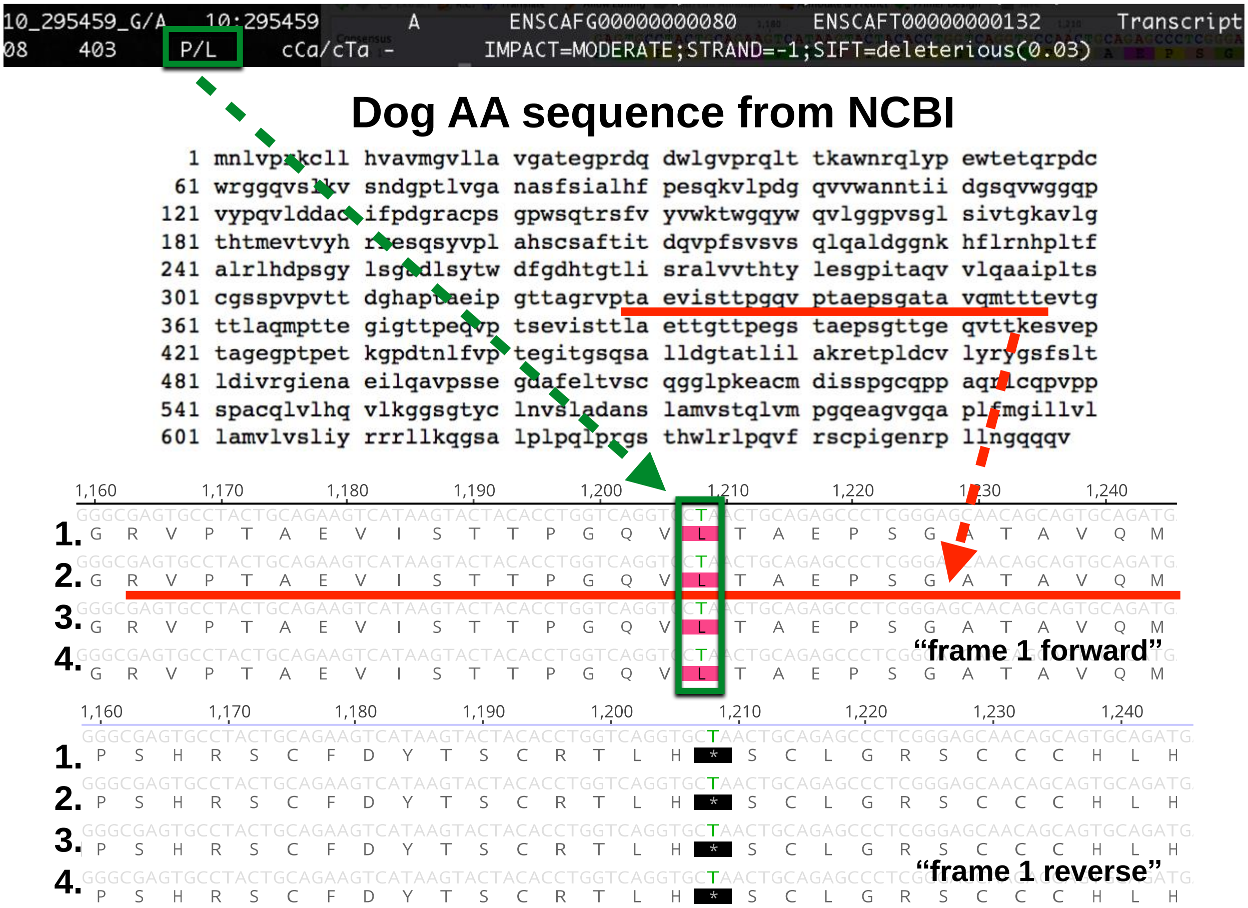

Figure S4. Translation of *PMEL* coding sequence with Geneious[24] under two orientations ("forward 1" vs "reverse 1"). Stop codon reported by ref.[19] is shown by an asterisk in the alignment with the incorrect reading frame ("frame 1 reverse" at the bottom) at position 1,023 bp, amino acid (AA) 341. The red arrow and line show the consistency of the correct reading frame ("forward 1" at the top) orientation with reported AA sequences in NCBI. The green arrow and rectangle indicates the concordance of the correct reading orientation ("forward 1") with the AA variation predicted by Variant Effect Predictor tool (black rectangle at the top). Numbers 1-4 indicate sequence identity among the four African wild dogs included in the analysis: 1. SAMN04312209, 2. RWK4881, 3. CN3669, and 4. SAMN04312208. The accession number for the NCBI dog AA sequence is NP_001096686.

MYO5A

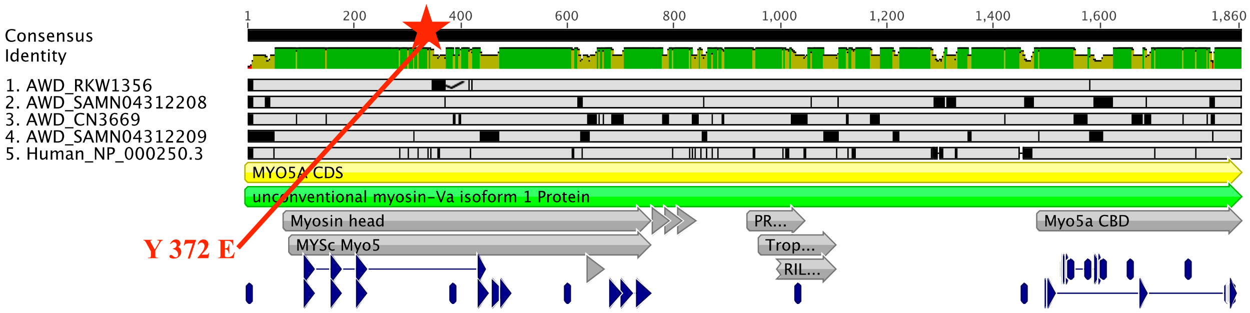

Figure S5. Protein alignments showing that the amino acid mutation unique in AWDs is located at the Myosin 5A head domain. Alignment between the four AWDs (sequences 1-4) and human (sequence 5). RWK481 and SAMN04312208 are individuals from Kruger National Park, South Africa; CN3669 and SAMN04312209 are individuals from Kenya. The red star indicates the amino acid mutation; protein domains are shown in gray.

Supplementary File

Configuration file for MCMCTree analyses in the PAML 4.8 package[11], including parameter settings for the clock model (global), substitution model (HKY85), birth-death process, gamma priors on the transition/transversion rate ratio (kappa_gamma) and shape parameter for variable rates among sites (alpha_gamma), the Dirichlet-gamma prior for the mean substitution rate (rgene_gamma), step sizes for proposals during the MCMC run, and the number of iterations, burnin and sample frequency of the MCMC.

```
seed = -1
seqfile = ../../Canids_othro_10_taxa.4fold_degenerate_sites.fasta
treefile = 2cal.tre
outfile = None

ndata = 1
seqtype = 0 * 0: nucleotides; 1:codons; 2:AAs
usedata = 1 * 0: no data; 1:seq like; 2:normal approximation; 3:out.BV (in.BV)
clock = 1 * 1: global clock; 2: independent rates; 3: correlated rates

model = 4 * 0:JC69, 1:K80, 2:F81, 3:F84, 4:HKY85
alpha = 0.500000 * alpha for gamma rates at sites
```

ncatG = 5 * No. categories in discrete gamma

cleandata = 0 * remove sites with ambiguity data (1:yes, 0:no)?

BDparas = 1.000000 1.000000 0.100000 * birth, death, sampling
kappa_gamma = 6.000000 2.000000 * gamma prior for kappa
alpha_gamma = 1.000000 1.000000 * gamma prior for alpha

rgene_gamma = 2.000000 2.000000 * gammaDir prior for rate for genes

finetune = 1: 0.100000 0.100000 0.100000 0.100000 0.100000 0.100000 * auto (0 or 1) : times, rates, mixing, paras, RateParas, FossilErr

print = 1 * write mcmc and summary to disk (mcmc.out)
burnin = 200000
sampfreq = 2
nsample = 2000000

References

1 Freedman, A. H. Genome Sequencing Highlights the Dynamic Early History of Dogs (vol 10, e1004016, 2014). *Plos Genetics* **10**, doi:10.1371/journal.pgen.1004631 (2014).

2 Fan, Z. X. *et al.* Worldwide patterns of genomic variation and admixture in gray wolves. *Genome Research* **26**, 163-173, doi:10.1101/gr.197517.115 (2016).

3 Langmead, B., Trapnell, C., Pop, M. & Salzberg, S. L. Ultrafast and memory-efficient alignment of short DNA sequences to the human genome. *Genome Biology* **10**, doi:10.1186/gb-2009-10-3-r25 (2009).

4 McKenna, A. *et al.* The Genome Analysis Toolkit: A MapReduce framework for analyzing next-generation DNA sequencing data. *Genome Research* **20**, 1297-1303, doi:10.1101/gr.107524.110 (2010).

5 Li, H. *et al.* The Sequence Alignment/Map format and SAMtools. *Bioinformatics* **25**, 2078-2079, doi:10.1093/bioinformatics/btp352 (2009).

6 Marsden, C. D. *et al.* Bottlenecks and selective sweeps during domestication have increased deleterious genetic variation in dogs. *Proceedings of the National Academy of Sciences of the United States of America* **113**, 152-157, doi:10.1073/pnas.1512501113 (2016).

7 Loytynoja, A. & Goldman, N. An algorithm for progressive multiple alignment of sequences with insertions. *Proceedings of the National Academy of Sciences of the United States of America* **102**, 10557-10562, doi:10.1073/pnas.0409137102 (2005).

8 Kumar, V. *et al.* The evolutionary history of bears is characterized by gene flow across species. *Scientific Reports* **7**, doi:10.1038/srep46487 (2017).

9 vonHoldt, B. M. *et al.* Whole-genome sequence analysis shows that two endemic species of North American wolf are admixtures of the coyote and gray wolf. *Science Advances* **2**, doi:10.1126/sciadv.1501714 (2016).
10 Gopalakrishnan, S. *et al.* Interspecific Gene Flow Shaped the Evolution of the Genus Canis. *Current Biology* **28**, 3441-3449, doi:10.1016/j.cub.2018.08.041 (2018).
11 Yang, Z. H. PAML 4: Phylogenetic analysis by maximum likelihood. *Molecular Biology and Evolution* **24**, 1586-1591, doi:10.1093/molbev/msm088 (2007).
12 Webb, A. E., Walsh, T. A. & O'Connell, M. J. VESPA: Very large-scale Evolutionary and Selective Pressure Analyses. *PeerJ Computer Science* **3**, 1-16 (2017).
13 Kaelin, C. B. & Barsh, G. S. Genetics of Pigmentation in Dogs and Cats. *Annual Review of Animal Biosciences* **1**, 125-156, doi:10.1146/annurev-animal-031412-103659 (2013).
14 Cooper, K. L. *et al.* Patterning and post-patterning modes of evolutionary digit loss in mammals. *Nature* **511**, 41-U537, doi:10.1038/nature13496 (2014).
15 Jernvall, J. & Thesleff, I. Reiterative signaling and patterning during mammalian tooth morphogenesis. *Mechanisms of Development* **92**, 19-29, doi:10.1016/s0925-4773(99)00322-6 (2000).
16 Daub, J. T., Moretti, S., Davydov, II, Excoffier, L. & Robinson-Rechavi, M. Detection of Pathways Affected by Positive Selection in Primate Lineages Ancestral to Humans. *Molecular Biology and Evolution* **34**, 1391-1402, doi:10.1093/molbev/msx083 (2017).
17 Koepfli, K. P. *et al.* Genome-wide Evidence Reveals that African and Eurasian Golden Jackals Are Distinct Species. *Current Biology* **25**, 2158-2165, doi:10.1016/j.cub.2015.06.060 (2015).
18 Auton, A. *et al.* Genetic Recombination Is Targeted towards Gene Promoter Regions in Dogs. *Plos Genetics* **9**, doi:10.1371/journal.pgen.1003984 (2013).
19 Campana, M. G. *et al.* Genome sequence, population history, and pelage genetics of the endangered African wild dog (Lycaon pictus). *Bmc Genomics* **17**, doi:10.1186/s12864-016-3368-9 (2016).
20 Gopalakrishnan, S. *et al.* Interspecific Gene Flow Shaped the Evolution of the Genus Canis. *Current Biology* **28**, 3441-+, doi:10.1016/j.cub.2018.08.041 (2018).
21 Armstrong, E. E. *et al.* Cost-effective assembly of the African wild dog (*Lycaon pictus*) genome using linked reads. (2018).
22 Robinson, J. A. *et al.* Genomic Flatlining in the Endangered Island Fox. *Current Biology* **26**, 1183-1189, doi:10.1016/j.cub.2016.02.062 (2016).
23 Skoglund, P., Ersmark, E., Palkopoulou, E. & Dalen, L. Ancient Wolf Genome Reveals an Early Divergence of Domestic Dog Ancestors and Admixture into High-Latitude Breeds. *Current Biology* **25**, 1515-1519, doi:10.1016/j.cub.2015.04.019 (2015).
24 Kearse, M. *et al.* Geneious Basic: An integrated and extendable desktop software platform for the organization and analysis of sequence data. *Bioinformatics* **28**, 1647-1649, doi:10.1093/bioinformatics/bts199 (2012).
